# Performance Analysis and Constellation Design for the Parallel Quadrature Spatial Modulation

**DOI:** 10.3390/e22080841

**Published:** 2020-07-30

**Authors:** Manar Mohaisen, Tasnim Holoubi, Tamer Abuhmed

**Affiliations:** 1 Department of Computer Science, Northeastern Illinois University, Chicago, IL 60625, USA; 2 Department of EEC Engineering, Korea Tech, Cheonan 31253, Korea; t.holoubi@gmail.com; 3 College of Computing, Sungkyunkwan University, Suwon 16419, Korea

**Keywords:** spatial modulation (SM), quadrature SM (QSM), parallel QSM (PQSM), constellation design, pairwise error probability

## Abstract

Spatial modulation (SM) is a multiple-input multiple-output (MIMO) technique that achieves a MIMO capacity by conveying information through antenna indices, while keeping the transmitter as simple as that of a single-input system. Quadrature SM (QSM) expands the spatial dimension of the SM into in-phase and quadrature dimensions, which are used to transmit the real and imaginary parts of a signal symbol, respectively. A parallel QSM (PQSM) was recently proposed to achieve more gain in the spectral efficiency. In PQSM, transmit antennas are split into parallel groups, where QSM is performed independently in each group using the same signal symbol. In this paper, we analytically model the asymptotic pairwise error probability of the PQSM. Accordingly, the constellation design for the PQSM is formulated as an optimization problem of the sum of multivariate functions. We provide the proposed constellations for several values of constellation size, number of transmit antennas, and number of receive antennas. The simulation results show that the proposed constellation outperforms the phase-shift keying (PSK) constellation by more than 10 dB and outperforms the quadrature-amplitude modulation (QAM) schemes by approximately 5 dB for large constellations and number of transmit antennas.

## 1. Introduction

Multiple-input multiple-output (MIMO) techniques, among others, strive to fulfill the ever increasing demands for high data rates in the future communication systems. These data rates requirements for the 5G, Beyond-5G, and 6G are 1 Gbps, 100 Gbps, and 1 Tbps, respectively [1]. Index modulation (IM) is a category of relatively new MIMO techniques that fulfills these high data requirements by allowing the transmission of information using the conventional signal symbols and the indices of given resources of the communication systems [2,3]. These indices represent antennas [4] or a combination of antennas [5], spreading codes [6], polarities [7], sub-carriers [8], or a combination of sub-carriers with multi-mode modulation [9,10], rotation angles [11], and virtual parallel channels [12], among others.

With the emergence of massive MIMO [13], spatial modulation (SM), in which signal symbols and the indices of the antennas used for transmission carry information, became a potential candidate for increasing the system capacity. An advantage of the SM system is that it requires a single radio-frequency (RF) chain. The SM is extended to the receiver side, where the index of a designated receive antenna carries information [14,15]. Macro-diversity precoding aided SM, where two base stations simultaneously communicate with a single user is proposed in Reference [16]. Quadrature SM (QSM) is an extension of the conventional SM that transmits a single signal symbol at each channel use, where the real and imaginary part is transmitted from the in-phase and quadrature spatial dimension, respectively [17]. The hardware implementation of the QSM using a single RF chain was investigated in Reference [18]. A precoding-aided QSM was proposed in Reference [19], where the indices of the designated receive antennas carry information. In transmit and receive SM, the system requires a large number of antennas to achieve high spectral efficiency. In addition to the cost and space requirements of installing more physical antennas, the channel estimation overhead is increased.

Building upon the SM, several systems that strike a trade-off between hardware requirements, i.e., the number of RF chains, and the achieved capacity were proposed. SM with multiple active antennas (MA-SM) transmits independent signal symbols from the activated antennas, leading to improvement in the system spectral efficiency [20]. The performance of the MA-SM is further investigated in Reference [21]. A special case of using two RF chains to simultaneously transmit two signal symbols at each channel use was proposed in Reference [22,23]. In this scenario, the first symbol is transmitted from a constellation set, and the second is transmitted from a rotated constellation. The rotation angle is optimized to reduce the error rate. To avoid transmitting the two signal symbols from the same antenna, an improved system equips the transmitter with an additional antenna that is used to transmit the second signal symbol only when the two symbols are supposed to be transmitted from the same antenna in the conventional system [24]. The constellation design of this system is investigated in Reference [25]. Another approach used to avoid the symbols’ overlapping and to reduce the number of transmit antennas by using antenna combinations is proposed in Reference [26]. Improved QSM (IQSM) exploits the in-phase and quadrature spatial dimensions to transmit the real and imaginary parts of two signal symbols, respectively, using combinations of two antennas [27]. The authors in Reference [28] investigate the constellation design of the IQSM and propose double QSM (DQSM) and parallel IQSM (PIQSM) that reduce the number of transmit antennas of the IQSM without additional hardware requirements. In addition to the information carried by the indices of the antennas used for transmission, the enhanced SM (ESM) conveys information through the constellation used to transmit either one or two signal symbols per channel use [29].

Another category of extensions split the set of transmit antennas into groups and perform any of the above SM techniques independently in each group. In Reference [30,31,32], SM is applied in each group, and the number of antennas per group is optimized. In Reference [33,34], QSM is performed in each group to increase the spectral efficiency of the system. Both systems achieve higher spectral efficiency compared to both SM and QSM, at the cost of requiring as many RF chains as the number of groups.

Another approach to improve the spectral efficiency and to reduce the number of antennas splits the available set of antennas into groups, where SM or QSM is performed in each antenna group using the same signal symbol. As such, the resulting transmitter design abides by the SM principal advantage of using a single RF chain while increasing the spectral efficiency at no cost at the transmitter side. The authors in Reference [35,36] proposed to perform the conventional SM in each group. This work was extended to the massive MIMO case in Reference [37]. The constellation design for this system with an arbitrary number of antenna groups is investigated in Reference [38]. To increase the spectral efficiency, an antenna grouping combined with QSM was recently proposed in Reference [39,40]. In the sequel, the systems in Reference [35,36,39,40] are referred to as parallel SM (PSM) and parallel QSM (PQSM), respectively.

**Contributions:** The contributions of this paper are as follows:

- The analytical upper-bound of the codeword pairwise error probability is derived for the PQSM with two and four groups.
- The derived upper-bound is formulated as a weighted sum of functions. We propose an improved constellation for the PQSM for several system configurations, where the search process is formulated as a multi-objective optimization problem. The obtained constellation reduces the asymptotic error performance, and it outperforms the conventional modulation schemes by more than 5 dB for given system configurations.

The rest of the paper is organized as follows: In Section 2, we present the system model and briefly describe several related works. In Section 3, the analytic performance of the PQSM is provided with QSM as a special case. The constellation design for the PQSM is introduced in Section 4. The simulation results are presented in Section 5, and the paper’s conclusions are drawn in Section 6.

## 2. System Model and Related Works

### 2.1. System Model

The assumed system consists of a transmitter equipped with $N_{t}$ transmit antennas and a receiver equipped with $N_{r}$ receive antennas. The number of transmit antennas is assumed to be a power of two; $N=\log_{2}\left(N_{t}\right)$, where *N* is an integer. The signal symbols are drawn from the modulation set A in which the size is $M=2^{q}$, where *q* is the number of bits per signal symbol. The elements of the channel matrix $H\in C^{N_{r}\times N_{t}}$ are assumed to be independent and identically distributed (i.i.d.) and follow a cyclically symmetric Gaussian distribution with mean and variance of zero and one, respectively. Only the receiver has a perfect knowledge of the channel matrix H. The noise is assumed to be additive white Gaussian with mean and variance of zero and $\sigma_{n}^{2}$, respectively.

### 2.2. Quadrature Spatial Modulation

**Figure 1:**
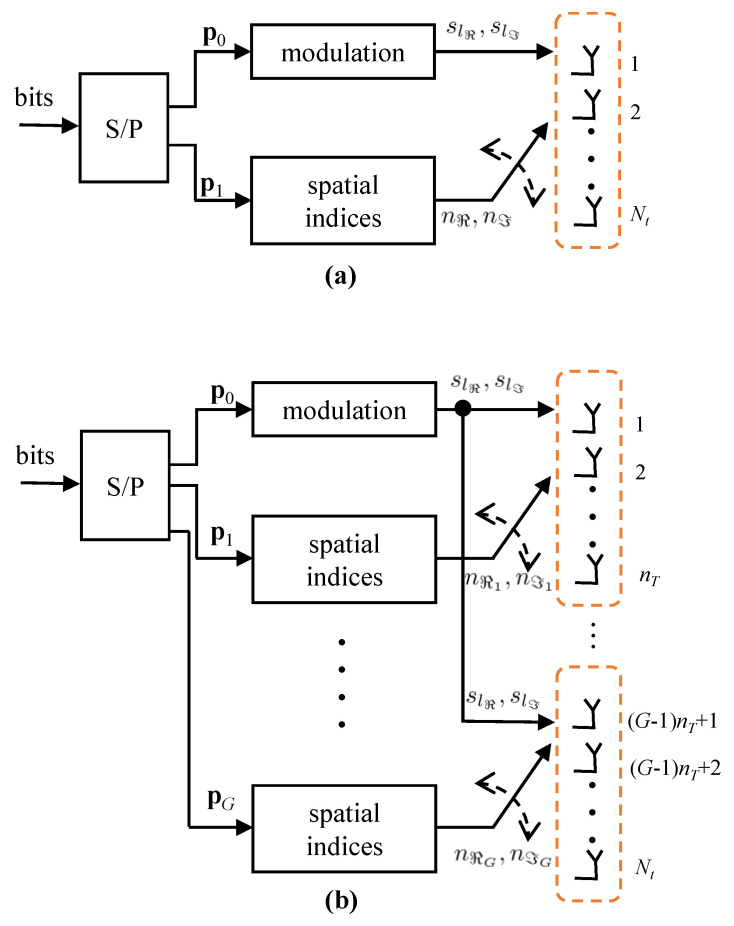
Simplified block diagram of (**a**) the conventional Quadrature Spacial Modulation (QSM) and (**b**) the parallel QSM (PQSM) with *G* antenna groups.

In QSM, the real and imaginary part of a signal symbol is transmitted from the in-phase and quadrature spatial dimension, respectively. The spectral efficiency of the QSM is K=(q+2N) bits per channel use (bpcu). Let $s_{l}=s_{l_{ℜ}}+js_{l_{ℑ}}$ be a signal symbol, the transmitted vector is given by:

(1) $$\begin{array}{c} s=e_{n_{ℜ}}s_{l_{ℜ}}+je_{n_{ℑ}}s_{l_{ℑ}}, \end{array}$$

where $e_{i}$ is the *i*th column of the $N_{t}$ identity matrix, and $n_{ℜ}$ and $n_{ℑ}$ are the indices of the antennas from which the real and imaginary parts of $s_{l}$ are, respectively, transmitted. Accordingly, the received signal vector is given by:

(2) $$y=\mathrm{Hs}+n=\left\{\begin{array}{cc} h_{n_{ℜ}}s_{l_{ℜ}}+jh_{n_{ℑ}}s_{l_{ℑ}}+n & \mathrm{if}\;n_{ℜ}\neq n_{ℑ}\\ h_{i}s_{l}+n & \mathrm{if}\;i=n_{ℜ}=n_{ℑ}, \end{array}\right.$$

where n is the additive white Gaussian noise. Figure 1a depicts a simplified block diagram of the conventional QSM.

Let the noiseless received vector be $g=h_{n_{ℜ}}s_{l_{ℜ}}+jh_{n_{ℑ}}s_{l_{ℑ}}$. The receiver employs the maximum-likelihood (ML) principle to recover $l,n_{ℜ}$ and $n_{ℑ}$ as follows:

(3) $$\begin{array}{cc} \left(\hat{l},{\hat{n}}_{ℜ},{\hat{n}}_{ℑ}\right) & =\underset{\begin{array}{c} l=1,\cdots ,M\\ n_{ℜ},n_{ℑ}=1,\cdots ,N_{t} \end{array}}{arg min}{∥y-g∥}^{2}\\ \; & =\underset{\begin{array}{c} l=1,\cdots ,M\\ n_{ℜ},n_{ℑ}=1,\cdots ,N_{t} \end{array}}{arg min}{∥g∥}^{2}-2ℜ\left\{y^{H}g\right\}, \end{array}$$

where ℜ{·} is the real part operator.

### 2.3. Parallel Quadrature Spatial Modulation

In PQSM, the available $N_{t}$ transmit antennas are divided into *G* disjoint groups, each of size $n_{T}=N_{t}/G$, where *G* is an integer and $2\leq G\leq N_{t}/2$. These groups are defined as follows:

(4) $${\left\{i\right\}}_{1}^{n_{T}},{\left\{i\right\}}_{n_{T}+1}^{2n_{T}},\cdots ,{\left\{i\right\}}_{\left(G-1\right)n_{T}+1}^{N_{t}}.$$

The conventional QSM is performed in each of these parallel groups using the same signal symbol $s_{l}$. The received vector is accordingly given by:

(5) $$\begin{array}{cc} y & =\frac{s_{l_{ℜ}}}{\sqrt{G}}\sum_{i=1}^{G}h_{n_{{ℜ}_{i}}}+j\frac{s_{l_{ℑ}}}{\sqrt{G}}\sum_{i=1}^{G}h_{n_{{ℑ}_{i}}}+n, \end{array}$$

where $n_{{ℜ}_{i}}$ and $n_{{ℑ}_{i}}$ are the indices of the antennas used for transmitting the real and imaginary parts of $s_{l}$ in the *i*th antenna group. Figure 1b depicts a simplified block diagram of the PQSM with *G* antenna groups. The normalization factor $1/\sqrt{G}$ is present in (5) to set the transmit power per signal symbol to unity.

The receiver employs the ML principle to recover the signal symbol and the vector of antenna indices as follows:

(6) $$\begin{array}{cc} \left(\hat{l},{\hat{n}}_{ℜ},{\hat{n}}_{ℑ}\right) & =\underset{l,n_{ℜ},n_{ℑ}}{arg min}{∥y-g∥}^{2}\\ \; & =\underset{l,n_{ℜ},n_{ℑ}}{arg min}{∥g∥}^{2}-2ℜ\left\{y^{H}g\right\}, \end{array}$$

where

(7) $$\begin{array}{cc} n_{ℜ} & =\left\{n_{{ℜ}_{1}},n_{{ℜ}_{2}},\cdots ,n_{{ℜ}_{G}}\right\}\\ n_{ℑ} & =\left\{n_{{ℑ}_{1}},n_{{ℑ}_{2}},\cdots ,n_{{ℑ}_{G}}\right\}. \end{array}$$

Note that the search in (3) is performed to find the index of the signal index *l* and the antenna indices $n_{ℜ}$ and $n_{ℑ}$ from which the real and imaginary parts of the signal symbol are transmitted. In (6), in addition to the index of the signal symbol, the ML detector searches for the sets $n_{ℜ}$ and $n_{ℑ}$, where $n_{{ℜ}_{i}}$ and $n_{{ℑ}_{i}}$ are the indices of the antennas from which the real and imaginary parts of the signal symbol are transmitted in the *i*-th antenna group.

Accordingly, the spectral efficiency of the PQSM is given by:

(8) $$K_{p}=q+2G\log_{2}\left(n_{T}\right).$$

Based on the above description, the spatial spectral efficiency, i.e., $2G\log_{2}\left(n_{T}\right)$, is a linear function in the number of antenna groups. A maximum spatial spectral efficiency can be achieved for $n_{T}=2$ and G = $N_{t}/2$. In this case, the spatial spectral efficiency is equal to $2G=N_{t}$ bpcu. The PQSM is explained through the following example.

**Example:** Let $N_{t}=8$, q=2, G=2. Assume that the codeword to be transmitted at a given channel use is

$$m=\left[0\;0\;1\;0\;1\;1\;0\;1\;0\;0\right].$$

The codeword is divided into the following (G+1) parts.

$$p_{0}=\left[0\;0\right],\;p_{1}=\left[\;1\;1\;0\;1\right],\;p_{2}=\left[0\;0\;1\;0\right],$$

where the length of $p_{0}$ is *q* bits and that of $p_{i}$, for i=1,⋯,G, is $2\log_{2}\left(n_{T}\right)$. The first two bits $p_{0}=\left[0\;0\right]$ modulate a signal symbol $s_{0}$ from a conventional modulation set. Let $s_{0}=\frac{1}{\sqrt{2}}-j\frac{1}{\sqrt{2}}$. The eight transmit antennas are split into two groups that contain the indices of the member antennas: $\left\{1,2,3,4\right\}$ and $\left\{5,6,7,8\right\}$. Assuming the first antenna group, the sequence $p_{1}$ is divided into two equal parts, where the first part modulates the index of the antenna from which the real part of $s_{0}$ is transmitted; the second part modulates the index of the antenna from which the imaginary part is transmitted. This yields $n_{{ℜ}_{1}}=2$ and $n_{{ℑ}_{1}}=4$. The conventional QSM is also performed using the same signal symbol $s_{0}$ in the second group, yielding $n_{{ℜ}_{2}}=7$ and $n_{{ℑ}_{2}}=5$. Mathematically, the transmitted symbols from the parallel groups are given by:

(9) $$\begin{array}{cc} s_{1} & =\frac{1}{\sqrt{2}}e_{2}-j\frac{1}{\sqrt{2}}e_{4}\\ s_{2} & =\frac{1}{\sqrt{2}}e_{7}-j\frac{1}{\sqrt{2}}e_{5}. \end{array}$$

Finally, the transmitted vector is given by $s={\left[s_{1}^{T}\;\;s_{2}^{T}\right]}^{T}/\sqrt{2}$ and the received vector is

(10) $$\begin{array}{c} y=\left(h_{2}+h_{7}\right)\frac{1}{2}-j\left(h_{4}+h_{5}\right)\frac{1}{2}+n. \end{array}$$

The ML principle in (7) is applied to recover the transmitted symbols.

## 3. Performance Analysis

### 3.1. Performance of the Quadrature Spatial Modulation

Let $g=h_{n_{ℜ}}s_{l_{ℜ}}+jh_{n_{ℑ}}s_{l_{ℑ}}$ and $g^{\prime }=h_{n_{ℜ}^{\prime }}s_{l_{ℜ}^{\prime }}+jh_{n_{ℑ}^{\prime }}s_{l_{ℑ}^{\prime }}$ be two received noiseless codewords, the pairwise error probability is then given by:

(11) $$\mathrm{Pr}\left[g\to g^{\prime }\right]=Q\left(\sqrt{\frac{\rho }{2}{∥g-g^{\prime }∥}^{2}}\right),$$

where $\rho =1/\sigma_{n}^{2}$ is the signal-to-noise ratio (SNR) and Q(·) is the Gaussian tail function, or simply the *Q*-function. The upper bound on the codeword pairwise error probability is obtained by summing over all possible pairs g and $g^{\prime }$ as follows:

(12) $$\mathrm{Pr}\left[e|H\right]\leq \sum_{n_{ℜ}=1}^{N_{t}}\sum_{n_{ℑ}=1}^{N_{t}}\sum_{n_{ℜ}^{\prime }=1}^{N_{t}}\sum_{n_{ℑ}^{\prime }=1}^{N_{t}}\sum_{l=1}^{M}\sum_{l^{\prime }=1}^{M}Q\left(\sqrt{\frac{\rho }{2}{∥h_{n_{ℜ}}s_{l_{ℜ}}+jh_{n_{ℑ}}s_{l_{ℑ}}-h_{n_{ℜ}^{\prime }}s_{l_{ℜ}^{\prime }}-jh_{n_{ℑ}^{\prime }}s_{l_{ℑ}^{\prime }}∥}^{2}}\right).$$

The union bound on the pairwise error probability is obtained by taking the expectation of both sides with respect to H [41,42]. We further simplify the asymptotic pairwise error probability as a weighted sum of multi-variate functions as follows [43]:

(13) Pr[e]≈2Nr−1Nrρ−NrM∑i=14fiΩi,

where $f_{1}={\left(N_{t}-1\right)}^{2}$, $f_{2}=f_{3}=\left(N_{t}-1\right)$ and $f_{4}=1$, and

(14) $$\begin{array}{c} \begin{array}{cccc} \Omega_{1} & =\sum_{l,l^{\prime }=1}^{M}\Lambda_{1}^{-N_{r}}=\sum_{l,l^{\prime }=1}^{M}{\left[s_{l_{ℜ}}^{2}+s_{l_{ℜ}^{\prime }}^{2}+s_{l_{ℑ}}^{2}+s_{l_{ℑ}^{\prime }}^{2}\right]}^{-N_{r}} & \; & \;\mathrm{if}\;n_{ℜ}\neq n_{ℜ}^{\prime }\;\mathrm{and}\;n_{ℑ}\neq n_{ℑ}^{\prime }\\ \Omega_{2} & =\sum_{l,l^{\prime }=1}^{M}\Lambda_{2}^{-N_{r}}=\sum_{l,l^{\prime }=1}^{M}{\left[{\left(s_{l_{ℜ}}-s_{l_{ℜ}^{\prime }}\right)}^{2}+s_{l_{ℑ}}^{2}+s_{l_{ℑ}^{\prime }}^{2}\right]}^{-N_{r}} & \; & \;\mathrm{if}\;n_{ℜ}=n_{ℜ}^{\prime }\;\mathrm{and}\;n_{ℑ}\neq n_{ℑ}^{\prime }\\ \Omega_{3} & =\sum_{l,l^{\prime }=1}^{M}\Lambda_{3}^{-N_{r}}=\sum_{l,l^{\prime }=1}^{M}{\left[s_{l_{ℜ}}^{2}+s_{l_{ℜ}^{\prime }}^{2}+{\left(s_{l_{ℑ}}-s_{l_{ℑ}^{\prime }}\right)}^{2}\right]}^{-N_{r}} & \; & \;\mathrm{if}\;n_{ℜ}\neq n_{ℜ}^{\prime }\;\mathrm{and}\;n_{ℑ}=n_{ℑ}^{\prime }\\ \Omega_{4} & =\sum_{\begin{array}{c} l,l^{\prime }=1\\ l\neq l^{\prime } \end{array}}^{M}\Lambda_{4}^{-N_{r}}=\sum_{\begin{array}{c} l,l^{\prime }=1\\ l\neq l^{\prime } \end{array}}^{M}{\left[{\left(s_{l_{ℜ}}-s_{l_{ℜ}^{\prime }}\right)}^{2}+{\left(s_{l_{ℑ}}-s_{l_{ℑ}^{\prime }}\right)}^{2}\right]}^{-N_{r}} & \; & \;\mathrm{if}\;n_{ℜ}=n_{ℜ}^{\prime }\;\mathrm{and}\;n_{ℑ}=n_{ℑ}^{\prime }. \end{array} \end{array}$$

The set $B_{\mathrm{qsm}}=\left\{\Lambda_{1},\Lambda_{2},\Lambda_{3},\Lambda_{4}\right\}$ is, by definition, the squared Euclidean distance at the transmitter between s and $s^{\prime }$ in the QSM system. Because the leading coefficients in (13) are fixed for given values of $N_{t}$, $N_{r}$ and ρ, the optimal modulation set for the QSM minimizes the term $\sum_{i=1}^{4}f_{i}\Omega_{i}$. To minimize $\Omega_{4}$, the Euclidean distance in the constellation should be maximized. M-ary quadrature-amplitude modulation (M-QAM) is conventionally designed to maximize the Euclidean distance. On the other hand, $\Omega_{1}$ does not depend on the distance among the signal symbols and is minimized if the power of the signal symbols is maximized under a transmission power constraint. The values of $f_{i}$ imply that at high values of $N_{t}$, $\Omega_{4}$ can be ignored. Accordingly, the *M* signal symbols are equally distributed over the four quadrants, where in each quadrant, the M/4 symbols are centered at the location of a standard QPSK symbol. In other words, the Euclidean distance among the symbols in each quadrant has a limited effect on the error performance for large $N_{t}$. This conjecture is valid only for small values of $N_{r}$. When $N_{r}$ takes large values, $\Omega_{4}$ becomes non-negligible as can be understood from (14).

### 3.2. Performance of the Parallel Quadrature Spatial Modulation

Let

(15) $$\begin{array}{cc} g & =s_{l_{ℜ}}\sum_{i=1}^{G}h_{n_{{ℜ}_{i}}}+js_{l_{ℑ}}\sum_{i=1}^{G}h_{n_{{ℑ}_{i}}},\\ g^{\prime } & =s_{l_{ℜ}^{\prime }}\sum_{i=1}^{G}h_{n_{{ℜ}_{i}}^{\prime }}+js_{l_{ℑ}^{\prime }}\sum_{i=1}^{G}h_{n_{{ℑ}_{i}}^{\prime }} \end{array}$$

be two received noiseless codewords. As in the case of the QSM, the pairwise error probability of the PQSM is obtained by substituting g and $g^{\prime }$ given in (15) into (11). Since the QSM is performed in parallel using independent channel matrices, the set containing the different expressions of the squared Euclidean distance between s and $s^{\prime }$ in the case of PQSM is given by:

(16) $$B_{\mathrm{pqsm},G}=\overset{G}{\underset{i=1}{⨁}}B_{\mathrm{qsm}},$$

where ⨁ denotes the Minkowski sum. We explain this process in light of the following example.

**Example**: Let $N_{t}=8$, G=2 and $n_{T}=N_{t}/G=4$ and B={1,3,4,6} with the frequencies {9,3,3,1}. Then,

(17) $$\begin{array}{cc} \overset{2}{\underset{i=1}{⨁}}B & =\{2,4,5,6,7,8,9,10,12\},\\ f & =\{81,54,54,9,36,9,6,6,1\}, \end{array}$$

where $\sum_{i}f_{i}={\left(n_{T}\right)}^{2G}=256$ in the above example. For G=2, the maximum number of elements of the Minkowski sum is equal to $\sum_{i=1}^{n_{T}}i=n_{T}\left(n_{T}+1\right)/2=10$.

The upper bound on the pairwise error probability of the PQSM is given by:

(18) Pr[e]≈2Nr−1Nrρ−NrM∑i=1BfiΩi,

where *B* is the number of unique Ω terms. Due to space limitations and the large size of $B_{\mathrm{pqsm},G}$ for large *G*, we restrict our analytic results to the case of G=2 and G=4.

For clarity, the expressions of $\Omega_{i}$ are given as a function in $\Lambda_{1}$ defined in (14). The nine Ω terms for the case of G=2 are given as follows:

(19) $$\begin{array}{cc} \Omega_{1} & =\sum_{l,l^{\prime }=1}^{M}{\left[2\Lambda_{1}\right]}^{-N_{r}}\\ \; & \;\mathrm{if}\;\left(n_{{ℜ}_{1}}\neq n_{{ℜ}_{1}}^{\prime }\;\mathrm{and}\;n_{{ℑ}_{1}}\neq n_{{ℑ}_{1}}^{\prime }\right)\;\mathrm{and}\;\left(n_{{ℜ}_{2}}\neq n_{{ℜ}_{2}}^{\prime }\;\mathrm{and}\;n_{{ℑ}_{2}}\neq n_{{ℑ}_{2}}^{\prime }\right)\\ \Omega_{2} & =\sum_{l,l^{\prime }=1}^{M}{\left[\Lambda_{1}+\Lambda_{2}\right]}^{-N_{r}}=\sum_{l,l^{\prime }=1}^{M}{\left[2\Lambda_{1}-2s_{l_{ℜ}}s_{l_{ℜ}^{\prime }}\right]}^{-N_{r}}\\ \; & \;\mathrm{if}\;\left(n_{{ℜ}_{1}}\neq n_{{ℜ}_{1}}^{\prime }\;\mathrm{and}\;n_{{ℑ}_{1}}\neq n_{{ℑ}_{1}}^{\prime }\right)\;\mathrm{and}\;\left(n_{{ℜ}_{2}}=n_{{ℜ}_{2}}^{\prime }\;\mathrm{and}\;n_{{ℑ}_{2}}\neq n_{{ℑ}_{2}}^{\prime }\right)\\ \; & \;\mathrm{or}\;\left(n_{{ℜ}_{1}}=n_{{ℜ}_{1}}^{\prime }\;\mathrm{and}\;n_{{ℑ}_{1}}\neq n_{{ℑ}_{1}}^{\prime }\right)\;\mathrm{and}\;\left(n_{{ℜ}_{2}}\neq n_{{ℜ}_{2}}^{\prime }\;\mathrm{and}\;n_{{ℑ}_{2}}\neq n_{{ℑ}_{2}}^{\prime }\right)\\ \Omega_{3} & =\sum_{l,l^{\prime }=1}^{M}{\left[\Lambda_{1}+\Lambda_{3}\right]}^{-N_{r}}=\sum_{l,l^{\prime }=1}^{M}{\left[2\Lambda_{1}-2s_{l_{ℑ}}s_{l_{ℑ}^{\prime }}\right]}^{-N_{r}}\\ \; & \;\mathrm{if}\;\left(n_{{ℜ}_{1}}\neq n_{{ℜ}_{1}}^{\prime }\;\mathrm{and}\;n_{{ℑ}_{1}}\neq n_{{ℑ}_{1}}^{\prime }\right)\;\mathrm{and}\;\left(n_{{ℜ}_{2}}\neq n_{{ℜ}_{2}}^{\prime }\;\mathrm{and}\;n_{{ℑ}_{2}}=n_{{ℑ}_{2}}^{\prime }\right)\\ \; & \;\mathrm{or}\;\left(n_{{ℜ}_{1}}\neq n_{{ℜ}_{1}}^{\prime }\;\mathrm{and}\;n_{{ℑ}_{1}}=n_{{ℑ}_{1}}^{\prime }\right)\;\mathrm{and}\;\left(n_{{ℜ}_{2}}\neq n_{{ℜ}_{2}}^{\prime }\;\mathrm{and}\;n_{{ℑ}_{2}}\neq n_{{ℑ}_{2}}^{\prime }\right)\\ \Omega_{4} & =\sum_{l,l^{\prime }=1}^{M}{\left[\Lambda_{1}+\Lambda_{4}\right]}^{-N_{r}}=\sum_{l,l^{\prime }=1}^{M}{\left[2\Lambda_{1}-2s_{l_{ℜ}}s_{l_{ℜ}^{\prime }}-2s_{l_{ℑ}}s_{l_{ℑ}^{\prime }}\right]}^{-N_{r}}\\ \; & \;\mathrm{if}\;\left(n_{{ℜ}_{1}}\neq n_{{ℜ}_{1}}^{\prime }\;\mathrm{and}\;n_{{ℑ}_{1}}\neq n_{{ℑ}_{1}}^{\prime }\right)\;\mathrm{and}\;\left(n_{{ℜ}_{2}}=n_{{ℜ}_{2}}^{\prime }\;\mathrm{and}\;n_{{ℑ}_{2}}=n_{{ℑ}_{2}}^{\prime }\right)\\ \; & \;\mathrm{or}\;\left(n_{{ℜ}_{1}}=n_{{ℜ}_{1}}^{\prime }\;\mathrm{and}\;n_{{ℑ}_{1}}=n_{{ℑ}_{1}}^{\prime }\right)\;\mathrm{and}\;\left(n_{{ℜ}_{2}}\neq n_{{ℜ}_{2}}^{\prime }\;\mathrm{and}\;n_{{ℑ}_{2}}\neq n_{{ℑ}_{2}}^{\prime }\right)\\ \; & \;\mathrm{or}\;\left(n_{{ℜ}_{1}}=n_{{ℜ}_{1}}^{\prime }\;\mathrm{and}\;n_{{ℑ}_{1}}\neq n_{{ℑ}_{1}}^{\prime }\right)\;\mathrm{and}\;\left(n_{{ℜ}_{2}}\neq n_{{ℜ}_{2}}^{\prime }\;\mathrm{and}\;n_{{ℑ}_{2}}=n_{{ℑ}_{2}}^{\prime }\right)\\ \; & \;\mathrm{or}\;\left(n_{{ℜ}_{1}}\neq n_{{ℜ}_{1}}^{\prime }\;\mathrm{and}\;n_{{ℑ}_{1}}=n_{{ℑ}_{1}}^{\prime }\right)\;\mathrm{and}\;\left(n_{{ℜ}_{2}}=n_{{ℜ}_{2}}^{\prime }\;\mathrm{and}\;n_{{ℑ}_{2}}\neq n_{{ℑ}_{2}}^{\prime }\right)\\ \Omega_{5} & =\sum_{l,l^{\prime }=1}^{M}{\left[2\Lambda_{2}\right]}^{-N_{r}}=\sum_{l,l^{\prime }=1}^{M}{\left[2\Lambda_{1}-4s_{l_{ℜ}}s_{l_{ℜ}^{\prime }}\right]}^{-N_{r}}\\ \; & \;\mathrm{if}\;\left(n_{{ℜ}_{1}}=n_{{ℜ}_{1}}^{\prime }\;\mathrm{and}\;n_{{ℑ}_{1}}\neq n_{{ℑ}_{1}}^{\prime }\right)\;\mathrm{and}\;\left(n_{{ℜ}_{2}}=n_{{ℜ}_{2}}^{\prime }\;\mathrm{and}\;n_{{ℑ}_{2}}\neq n_{{ℑ}_{2}}^{\prime }\right)\\ \Omega_{6} & =\sum_{l,l^{\prime }=1}^{M}{\left[\Lambda_{2}+\Lambda_{4}\right]}^{-N_{r}}=\sum_{l,l^{\prime }=1}^{M}{\left[2\Lambda_{1}-4s_{l_{ℜ}}s_{l_{ℜ}^{\prime }}-2s_{l_{ℜ}}s_{l_{ℜ}^{\prime }}\right]}^{-N_{r}}\\ \; & \;\mathrm{if}\;\left(n_{{ℜ}_{1}}=n_{{ℜ}_{1}}^{\prime }\;\mathrm{and}\;n_{{ℑ}_{1}}\neq n_{{ℑ}_{1}}^{\prime }\right)\;\mathrm{and}\;\left(n_{{ℜ}_{2}}=n_{{ℜ}_{2}}^{\prime }\;\mathrm{and}\;n_{{ℑ}_{2}}=n_{{ℑ}_{2}}^{\prime }\right)\\ \; & \;\mathrm{or}\;\left(n_{{ℜ}_{1}}=n_{{ℜ}_{1}}^{\prime }\;\mathrm{and}\;n_{{ℑ}_{1}}=n_{{ℑ}_{1}}^{\prime }\right)\;\mathrm{and}\;\left(n_{{ℜ}_{2}}=n_{{ℜ}_{2}}^{\prime }\;\mathrm{and}\;n_{{ℑ}_{2}}\neq n_{{ℑ}_{2}}^{\prime }\right)\\ \Omega_{7} & =\sum_{l,l^{\prime }=1}^{M}{\left[2\Lambda_{3}\right]}^{-N_{r}}=\sum_{l,l^{\prime }=1}^{M}{\left[2\Lambda_{1}-4s_{l_{ℑ}}s_{l_{ℑ}^{\prime }}\right]}^{-N_{r}}\\ \; & \;\mathrm{if}\;\left(n_{{ℜ}_{1}}\neq n_{{ℜ}_{1}}^{\prime }\;\mathrm{and}\;n_{{ℑ}_{1}}=n_{{ℑ}_{1}}^{\prime }\right)\;\mathrm{and}\;\left(n_{{ℜ}_{2}}\neq n_{{ℜ}_{2}}^{\prime }\;\mathrm{and}\;n_{{ℑ}_{2}}=n_{{ℑ}_{2}}^{\prime }\right)\\ \Omega_{8} & =\sum_{l,l^{\prime }=1}^{M}{\left[\Lambda_{3}+\Lambda_{4}\right]}^{-N_{r}}=\sum_{l,l^{\prime }=1}^{M}{\left[2\Lambda_{1}-2s_{l_{ℜ}}s_{l_{ℜ}^{\prime }}-4s_{l_{ℑ}}s_{l_{ℑ}^{\prime }}\right]}^{-N_{r}}\\ \; & \;\mathrm{if}\;\left(n_{{ℜ}_{1}}\neq n_{{ℜ}_{1}}^{\prime }\;\mathrm{and}\;n_{{ℑ}_{1}}=n_{{ℑ}_{1}}^{\prime }\right)\;\mathrm{and}\;\left(n_{{ℜ}_{2}}=n_{{ℜ}_{2}}^{\prime }\;\mathrm{and}\;n_{{ℑ}_{2}}=n_{{ℑ}_{2}}^{\prime }\right)\\ \; & \;\mathrm{or}\;\left(n_{{ℜ}_{1}}=n_{{ℜ}_{1}}^{\prime }\;\mathrm{and}\;n_{{ℑ}_{1}}=n_{{ℑ}_{1}}^{\prime }\right)\;\mathrm{and}\;\left(n_{{ℜ}_{2}}\neq n_{{ℜ}_{2}}^{\prime }\;\mathrm{and}\;n_{{ℑ}_{2}}=n_{{ℑ}_{2}}^{\prime }\right)\\ \Omega_{9} & =\sum_{\begin{array}{c} l,l^{\prime }=1\\ l\neq l^{\prime } \end{array}}^{M}{\left[2\Lambda_{4}\right]}^{-N_{r}}=\sum_{\begin{array}{c} l,l^{\prime }=1\\ l\neq l^{\prime } \end{array}}^{M}{\left[2{\left(s_{l_{ℜ}}-s_{l_{ℜ}^{\prime }}\right)}^{2}+2{\left(s_{l_{ℑ}}-s_{l_{ℑ}^{\prime }}\right)}^{2}\right]}^{-N_{r}}\\ \; & =\sum_{\begin{array}{c} l,l^{\prime }=1\\ l\neq l^{\prime } \end{array}}^{M}{\left[2\Lambda_{1}-4s_{l_{ℜ}}s_{l_{ℜ}^{\prime }}-4s_{l_{ℑ}}s_{l_{ℑ}^{\prime }}\right]}^{-N_{r}}\\ \; & \;\mathrm{if}\;\left(n_{{ℜ}_{1}}=n_{{ℜ}_{1}}^{\prime }\;\mathrm{and}\;n_{{ℑ}_{1}}=n_{{ℑ}_{1}}^{\prime }\right)\;\mathrm{and}\;\left(n_{{ℜ}_{2}}=n_{{ℜ}_{2}}^{\prime }\;\mathrm{and}\;n_{{ℑ}_{2}}=n_{{ℑ}_{2}}^{\prime }\right). \end{array}$$

The frequencies of these terms are given by:

(20) $$\begin{array}{cc} f_{1} & ={\left(n_{T}-1\right)}^{4},f_{2}=f_{3}=2{\left(n_{T}-1\right)}^{3},\\ f_{4} & =4{\left(n_{T}-1\right)}^{2},f_{5}=f_{7}={\left(n_{T}-1\right)}^{2},\\ f_{6} & =f_{8}=2\left(n_{T}-1\right),f_{9}=1. \end{array}$$

Note that $B_{\mathrm{pqsm},G}={⨁}_{i=1}^{2}B_{\mathrm{pqsm},G/2}$.

The elements of the set $B_{\mathrm{pqsm},4}$ and the corresponding frequencies in the case of G=4 are listed in Table 1. In Table 1, $\Lambda_{1}=\left[s_{l_{ℜ}}^{2}+s_{l_{ℜ}^{\prime }}^{2}+s_{l_{ℑ}}^{2}+s_{l_{ℑ}^{\prime }}^{2}\right]$ as defined in (14), $\Lambda_{ℜ}=s_{l_{ℜ}}s_{l_{ℜ}}^{\prime }$, $\Lambda_{ℑ}=s_{l_{ℑ}}s_{l_{ℑ}}^{\prime }$, and $n=n_{T}-1$. Any of the 25 Ω terms is obtained by substituting the corresponding Λ term in $\sum_{l,l^{\prime }=1}^{M}{\left[\Lambda \right]}^{-N_{r}}$. For instance,

(21) $$\Omega_{2}=\sum_{l,l^{\prime }=1}^{M}{\left[4\Lambda_{1}-2\Lambda_{ℜ}-2\Lambda_{ℑ}\right]}^{-N_{r}}.$$

The optimization of the modulation set for the PQSM is addressed in the next section.

## 4. Constellation Design for the PQSM

The constellation set is optimized to reduce the asymptotic pairwise error probability given in (18). For a given $N_{t}$, $N_{r}$, *M*, and *G*, the optimization of the modulation set is given as follows:

(22) $$\begin{array}{c} \underset{-\sqrt{M}\leq s_{l_{ℜ}},s_{l_{ℑ}}\leq \sqrt{M}}{arg min}\left(\sum_{i=1}^{B}f_{i}\Omega_{i}\right),\\ \;s.t.\;\sum_{l=1}^{M}\left(s_{l_{ℜ}}^{2}+s_{l_{ℑ}}^{2}\right)=M. \end{array}$$

where B=4, 9, and 25 in the PQSM with G=1,2, and 4, respectively. Obtaining an analytic solution for the above optimization problem is difficult. Therefore, we used the optimization toolbox of MATLAB to obtain the solution numerically. This optimization process does not depend on the channel or noise realization and, therefore, can be done offline. We follow the conventional design of constellation sets, where the signal symbols are equally distributed among the four Euclidean space quadrants and are symmetric over the in-phase and quadrature axes. To avoid locating the real or imaginary part of the signal symbol on the in-phase or quadrature axis, respectively, we set a lower-bound value for the real and imaginary parts. We refer to this value by the positive number α. Accordingly, the multi-objective optimization of (18) is simplified as follows:

(23) $$\begin{array}{cc} \; & \underset{\begin{array}{c} \alpha \leq s_{l_{ℜ}},s_{l_{ℑ}}\leq \sqrt{M}\\ l=1,\cdots ,M/4 \end{array}}{arg min}\sum_{i=1}^{B}f_{i}\Omega_{i},\\ \; & \;s.t.\;\sum_{l=1}^{M/4}{\left|s_{l}\right|}^{2}=M/4. \end{array}$$

The value of α is set to 0.1 through simulations: Smaller values might reduce the Euclidean distance among symbols located in different quadrants, and larger values might reduce the Euclidean distance among signal symbols within each quadrant. Note that the obtained symbols by solving (23) are located in the first quadrant, and the remaining 3/4M symbols are obtained following the rule of symmetry over the in-phase and quadrature axes.

**Figure 2:**
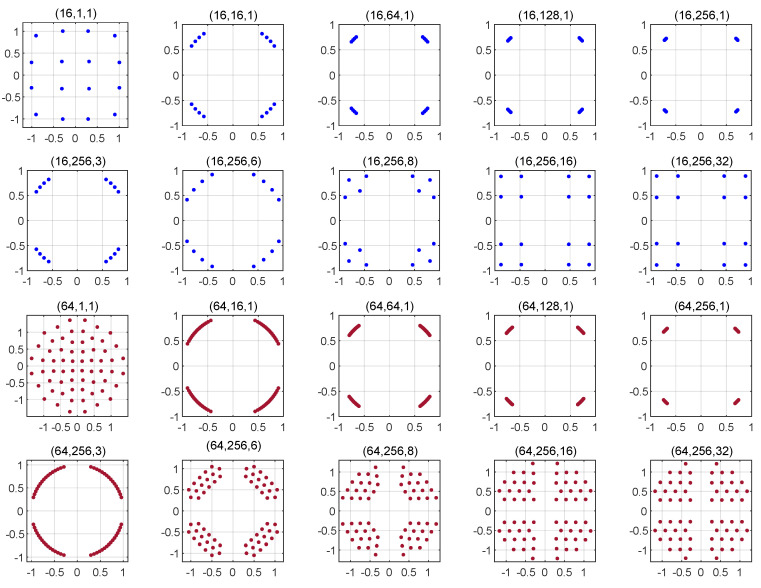
Optimal modulation set for the PQSM for several combinations of $\left(M,n_{T},N_{r}\right)$ and G=2.

Figure 2 depicts the proposed constellation for M=16, 64 and several values of $n_{T}$ and $N_{r}$. The first row of sub-figures depict the proposed constellation for M=16, $N_{r}=1$ and several values of $n_{T}$, whereas the second row of sub-figures depict the proposed constellation for fixed M=16 and $n_{T}=256$, and several values of $N_{r}$. The third and fourth rows depict the proposed constellations for the M=64 and a variable number of transmit and receive antennas. Based on these results, we make the following remarks.

**Figure 3:**
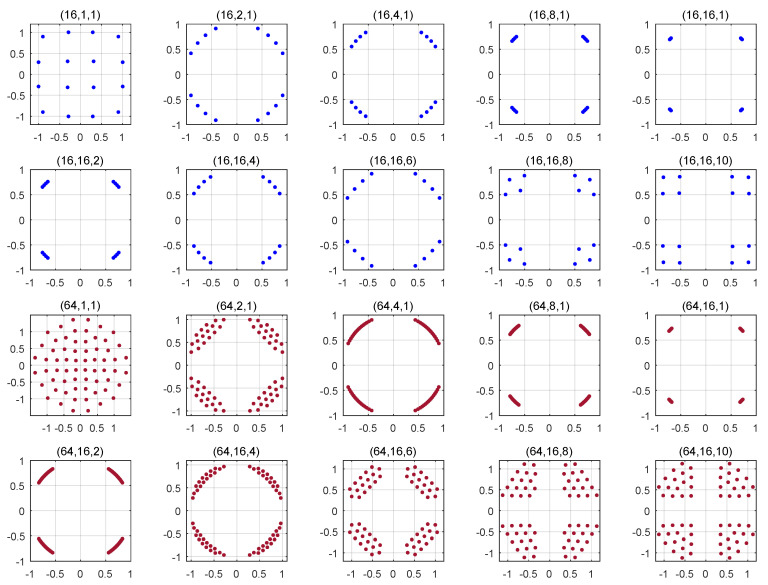
Optimal modulation set for the PQSM for several combinations of $\left(M,n_{T},N_{r}\right)$ and G=4.

- The nine Ω terms given in (19) can be split into three groups. The first group consists of $\Omega_{1}$. To minimize this term, the energy of the symbols should be maximized under the transmission power constraint; the average power per symbol is equal to one. Therefore, $\Omega_{1}$ is minimized if all the symbols have an equal power of one. Based on the design constraint mentioned above, where M/4 symbols are located in each of the four quadrants, the M/4 symbols will be located at the location of a QPSK symbol. The term $\Lambda_{1}$ is referred to as the energy-maximization term. The second group consists of $\Omega_{9}$. To minimize this term, the Euclidean distance between the signal symbols should be maximized. Under the transmission power constraint, this leads to a constellation similar to the standard quadrature amplitude modulation (QAM) set. The term $\Lambda_{4}$ is referred to as the distance-maximization term. The third group consists of the remaining terms $\Omega_{2}$ to $\Omega_{8}$. These terms are combinations of the energy- and distance-maximization terms. The result of the disjoint optimization of these terms strikes a trade-off between maximizing the energy of the symbols and increasing the Euclidean distance among them. In light of (19), maximizing the energy will reduce the Ω terms more than maximizing the distance among the symbols does.
- For a fixed and relatively small number of receive antennas $N_{r}$, the energy-maximization term dominates the optimization process. This is supported by the tendency of the proposed constellations depicted in the first and third rows of Figure 2: The symbols of the proposed constellation are located at the location of the standard QPSK symbols.
- As $N_{r}$ increases, $\Omega_{9}$ also increases as it is the reciprocal of the Euclidean distance between the real parts and imaginary parts raised to a power of $N_{r}$. To reduce the pairwise error probability, the Euclidean distance among the symbols should be increased. That is why the obtained constellation for high $N_{r}$ is a QAM-like modulation set. This is very clear in the case of M=16, and the Euclidean distance among the symbols in the obtained constellation increases for M=64 for high $N_{r}$.
- Figure 3 depicts the proposed constellation for M=16 and 64 with G=4. The analysis given above for G=2 is still valid for G=4. The only remarkable difference between the two scenarios is that as *G* increases, the shape of the proposed constellation converges to the standard QPSK more rapidly as a function of $n_{T}$. This convergence tendency of the proposed constellation as a function of $n_{T}$ is due to the low weight associated with the distance-maximizing term as *G* increases.

## 5. Simulation Results and Discussion

In this section, we assume that the channel coefficients are independent and follow a centered circularly-symmetric complex Gaussian distribution with mean and variance of 0 and 1, respectively. The channel state information is assumed to be known only at the receiver. For all the evaluated systems, we assume that the receiver employs the maximum-likelihood principle to jointly recover both the signal and spatial symbols.

**Figure 4:**
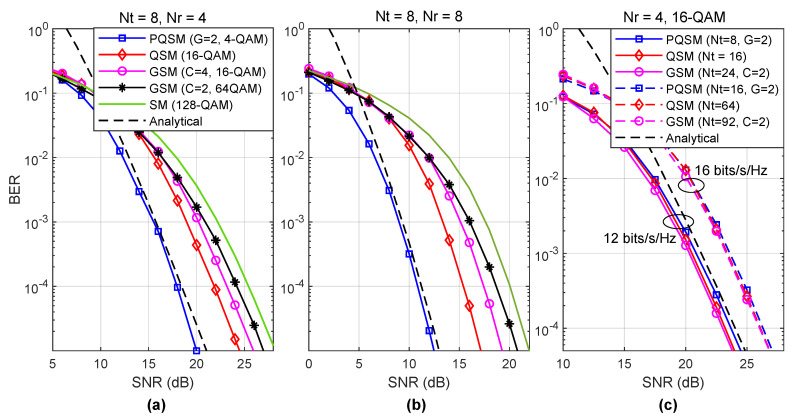
Comparison among PQSM, QSM, generalized SM (GSM), and SM for the same spectral efficiency: (**a**) $N_{t}=8,N_{r}=4$ and different modulation orders adjusted to achieve a fixed spectral efficiency, (**b**) $N_{t}=N_{r}=8$, and (**c**) spectral efficiency of 12 and 16 bits/s/Hz, $N_{r}=4$, and different values of $N_{t}$.

Figure 4 depicts a comparison between the PQSM for G=2, SM, GSM, and QSM systems. The simulation results depicted in Figure 4a are obtained for fixed $N_{t}=8$ and $N_{r}=4$ and a variable modulation order for different systems. The modulation order is set to guarantee an equal total spectral efficiency of 10 bits/s/Hz for the four compared systems. The performance of the generalized SM (GSM) is depicted using antenna combinations of length C=2 and C=4. At a target bit-error-rate (BER) of $10^{-4}$, PQSM with G=2 outperforms QSM, GSM with C=4, GSM with C=2, and SM by approximately 4, 5, 6, and 7.5 dB, respectively. To evaluate the effect of $N_{r}$ on the gain achieved by the PQSM compared to the conventional systems, Figure 4b depicts the performance of the four systems using the same parameters depicted in Figure 4a with $N_{r}=8$. At a target BER of $10^{-4}$, PQSM with G=2 outperforms QSM, GSM with C=4, GSM with C=2, and SM by approximately 4.8, 6.5, 7.5, and 9.4 dB, respectively. Finally, the performances of PQSM, QSM and GSM for spectral efficiencies of 12 and 16 bits/s/Hz are depicted in Figure 4c, where all systems use 16-QAM and $N_{r}=4$. The number of transmit antennas for each of the three systems is selected to unify the achieved spectral efficiency. While the gap in the BER performance among the three systems for the two scenarios is negligible, PQSM requires a small fraction of the number of antennas required by the other two systems. For instance, PQSM requires 16 antennas, whereas QSM and GSM require 64 and 92 antennas, respectively, to achieve a spectral efficiency of 16 bits/s/Hz. These comparisons reflect the superior performance of the PQSM and its suitability for future communication systems. Accordingly, the evaluation of a dedicated design of the constellation set for the PQSM is analyzed in the following. The analytical results of the PQSM for the different scenarios are also provided in Figure 4. The analytical results are an upper-bound on the error performance for high SNR.

**Figure 5:**
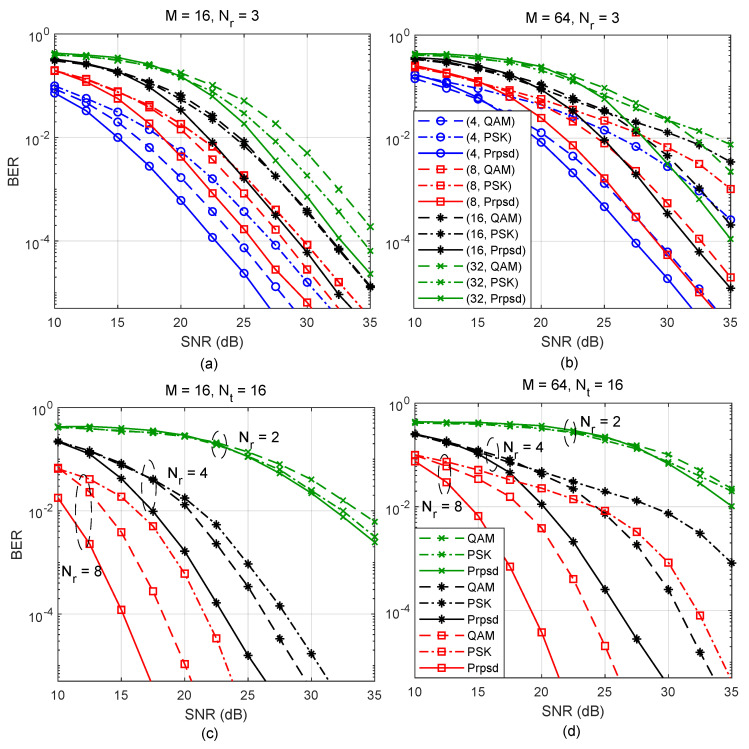
Simulation results of the the PQSM with M-ary quadrature-amplitude modulation (M-QAM), M-ary Phase-Shift Keying (M-PSK) and the proposed constellations, and G=2. (**a**) M=16, $N_{r}=3$, and $N_{t}=4,8,16$, and 32, (**b**) M=64, $N_{r}=3$, and $N_{t}=4,8,16$, and 32, (**c**) M=16, $N_{t}=16$, and $N_{r}=2,4$, and 8, (**d**) M=64, $N_{t}=16$, and $N_{r}=2,4$, and 8.

Figure 5 depicts a comparison of the performance of the PQSM using the conventional QAM and PSK constellations versus the proposed constellation, for G=2. Figure 5a shows the performance of the PQSM for M=16, $N_{r}=3$, and $N_{t}=4,8,16,$ and 32. For $N_{t}=4$, the proposed constellation outperforms QAM and PSK by 1.8 and 4.3 dB, respectively. For $N_{t}=8$, the proposed constellation outperforms QAM and PSK by 2.3 and 4.0 dB, respectively. For $N_{t}=16$, the proposed constellation outperforms QAM and PSK by 2.8 and 2.7 dB, respectively. For $N_{t}=32$, the proposed constellation outperforms QAM and PSK by 3.1 and 1.6 dB, respectively. For small values of $N_{t}$, the proposed constellation is similar to the conventional QAM set. Therefore, the performance gap between the proposed constellation and that of QAM is relatively small. On the other hand, PSK constellation has worse performance compared to QAM and the proposed constellation. As $N_{t}$ becomes large, the proposed constellation is more similar to PSK set, and the performance gap between the two constellations is reduced. The performance of QAM constellation degrades for large values of $N_{t}$. These results and analysis are consistent with the results depicted in Figure 2. Figure 5b depicts the system’s performance for M=64. At a target BER of $10^{-4}$, the performance of the proposed constellation outperforms QAM and PSK sets by 3.5 and 10 dB, respectively, for $N_{t}=8$. The gain achieved by the proposed constellation increases for larger values of $N_{t}$.

Figure 5c,d depict the performance of the proposed constellation for M=16 and M=64, respectively, for a fixed $N_{t}$ and $N_{r}=2$, 4, and 8. For $N_{r}=8$ and M=16, the proposed constellation outperforms QAM and PSK by 3 and 7.4 dB, respectively. The performance gain achieved by the proposed constellation over QAM and PSK increases to 4.5 and 13 dB, respectively, for M=64.

**Figure 6:**
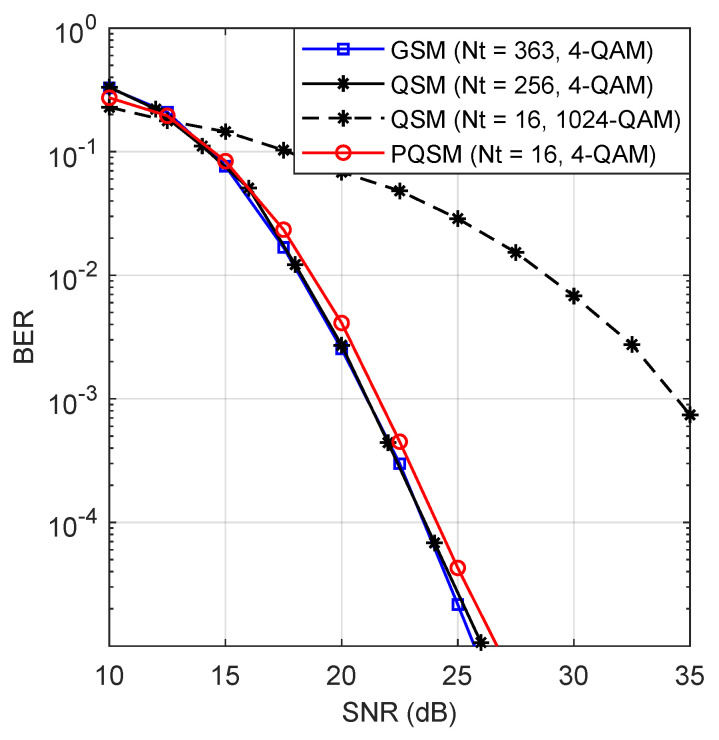
Simulation results comparison between GSM (C=2), QSM, and PQSM for G=4 for a spectral efficiency of 18 bits/s/Hz.

Figure 6 depicts the performance of the PQSM for G=4 and those of GSM and QSM. To achieve a similar error performance and the same spectral efficiency, PQSM requires 16 antennas, while GSM and QSM require 363 and 256 antennas, respectively. To reduce the number of transmit antennas in the case of QSM, a high order modulation is required to achieve the same spectral efficiency. This comes at a heavy cost in terms of the error performance. For instance, PQSM outperforms QSM by approximately 12 dB at a target BER of $10^{-3}$, when both systems use the same $N_{t}=16$.

**Figure 7:**
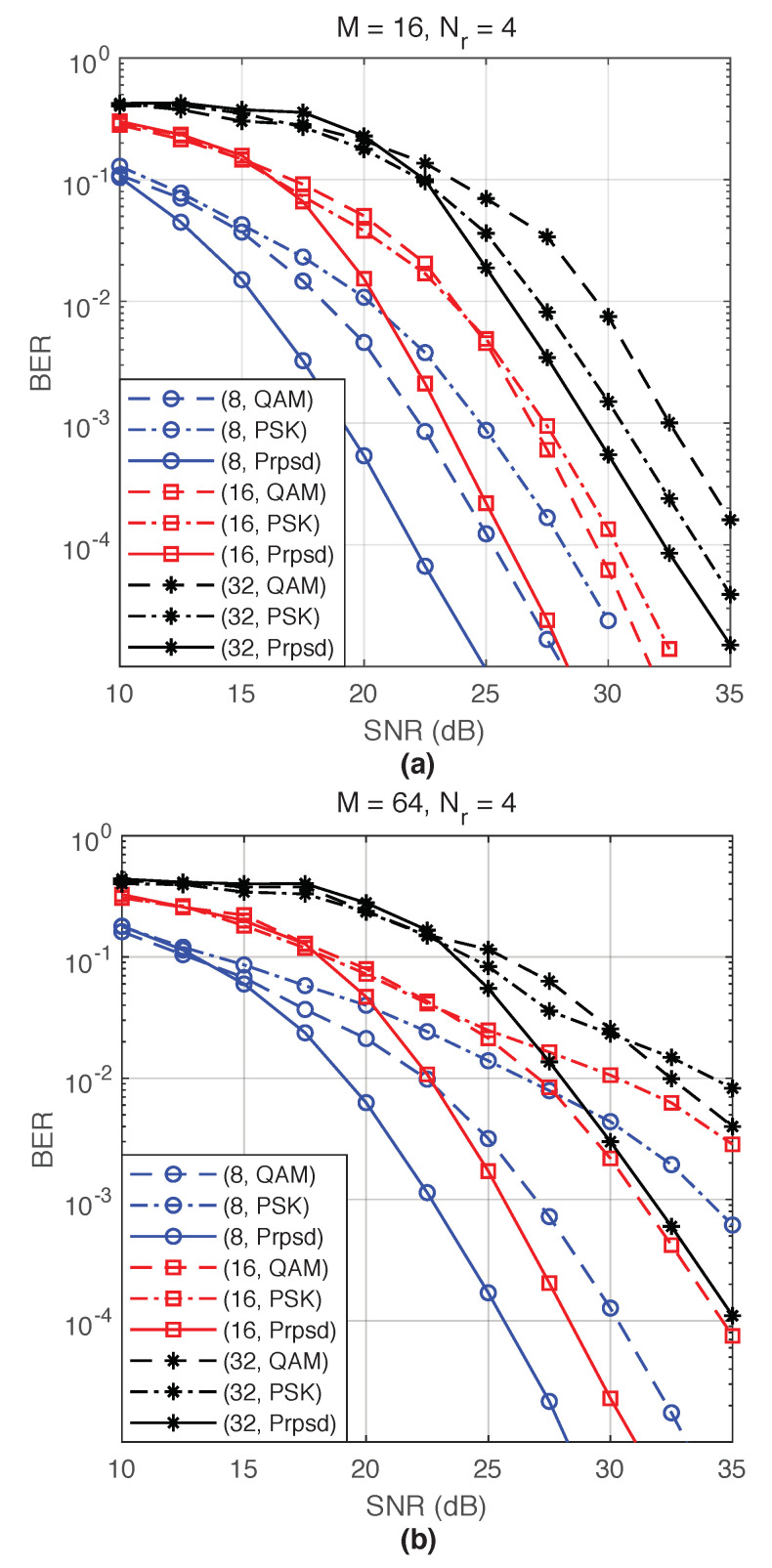
Simulation results of the the PQSM with M-QAM, M-PSK and the proposed constellations, and G=4. (**a**) M=16, $N_{r}=4$, and $N_{t}=8,16$, and 32, and (**b**) M=64, $N_{r}=4$, and $N_{t}=8,16$, and 32.

Finally, the performance of the proposed constellation is compared to those of the conventional PSK and QAM for G=4. In Figure 7a, the proposed constellation outperforms QAM and PSK by 3.5 and 6 dB, respectively, for M=16. For a large value of $N_{t}$, the proposed constellation’s shape is more similar to the PSK than QAM. This explains the performance of the three constellations at $N_{t}=32$ in Figure 7, which shows the performance for M=64. At a target BER of $10^{-3}$, the proposed constellation outperforms QAM and PSK by 4 and 11.5 dB, respectively, for $N_{t}=8$. These results demonstrate the merits of the proposed constellation.

Recently, most wide-band communication systems use orthogonal-frequency division multiplexing (OFDM) as the radio technology due to its proven merits. Inspired by the works in Reference [4,8,44], we would like investigate the performance of combining PQSM with OFDM in SM-OFDM and index-modulation-OFDM (IM-OFDM) scenarios. While the combination of PQSM and SM-OFDM is straightforward, the implementation of the PQSM on top of the IM-OFDM system requires careful consideration and possible modifications to the PQSM technique.

## 6. Conclusions

The PQSM is a recent technique that achieves high spectral efficiency through expanding the spatial dimensions over which a single signal is transmitted. Conventionally, M-ary PSK or QAM constellations are used to modulate the signal symbols. In this paper, we derived the asymptotic pairwise error probability of the PQSM and formulated it as a sum of weighted multi-variate functions. Exact analytical results of these functions are provided for two and four parallel groups. The search for the optimal constellation is formulated as an optimization problem that reduces the asymptotic pairwise error probability. We discussed the tendency of the shape of the proposed constellation for an arbitrary number of parallel antenna groups. The simulation results show that the proposed constellation achieves as high as 10 dB of SNR gain over the conventional modulation schemes.

## Figures and Tables

**Table 1:** The Λ terms used to evaluated the pairwise error probability of the PQSM for G=4.

	Λ	*f*
1	$4\Lambda_{1}$	$n^{8}$
2	$4\Lambda_{1}-2\Lambda_{ℜ}-2\Lambda_{ℑ}$	$16n^{6}$
3	$4\Lambda_{1}-4\Lambda_{ℜ}-4\Lambda_{ℑ}$	$36n^{4}$
4	$4\Lambda_{1}-6\Lambda_{ℜ}-6\Lambda_{ℑ}$	$16n^{2}$
5	$4\Lambda_{1}-8\Lambda_{ℜ}-8\Lambda_{ℑ}$	1
6	$4\Lambda_{1}-2\Lambda_{ℜ}$	$4n^{7}$
7	$4\Lambda_{1}-2\Lambda_{ℑ}$	$4n^{7}$
8	$4\Lambda_{1}-4\Lambda_{ℜ}$	$6n^{6}$
9	$4\Lambda_{1}-4\Lambda_{ℑ}$	$6n^{6}$
10	$4\Lambda_{1}-4\Lambda_{ℜ}-2\Lambda_{ℑ}$	$24n^{5}$
11	$4\Lambda_{1}-2\Lambda_{ℜ}-4\Lambda_{ℑ}$	$24n^{5}$
12	$4\Lambda_{1}-6\Lambda_{ℜ}$	$4n^{5}$
13	$4\Lambda_{1}-6\Lambda_{ℑ}$	$4n^{5}$
14	$4\Lambda_{1}-6\Lambda_{ℜ}-2\Lambda_{ℑ}$	$16n^{4}$
15	$4\Lambda_{1}-2\Lambda_{ℜ}-6\Lambda_{ℑ}$	$16n^{4}$
16	$4\Lambda_{1}-6\Lambda_{ℜ}-4\Lambda_{ℑ}$	$24n^{3}$
17	$4\Lambda_{1}-4\Lambda_{ℜ}-6\Lambda_{ℑ}$	$24n^{3}$
18	$4\Lambda_{1}-8\Lambda_{ℜ}$	$n^{4}$
19	$4\Lambda_{1}-8\Lambda_{ℑ}$	$n^{4}$
20	$4\Lambda_{1}-8\Lambda_{ℜ}-2\Lambda_{ℑ}$	$4n^{3}$
21	$4\Lambda_{1}-2\Lambda_{ℜ}-8\Lambda_{ℑ}$	$4n^{3}$
22	$4\Lambda_{1}-8\Lambda_{ℜ}-4\Lambda_{ℑ}$	$6n^{2}$
23	$4\Lambda_{1}-4\Lambda_{ℜ}-8\Lambda_{ℑ}$	$6n^{2}$
24	$4\Lambda_{1}-8\Lambda_{ℜ}-6\Lambda_{ℑ}$	4n
25	$4\Lambda_{1}-6\Lambda_{ℜ}-8\Lambda_{ℑ}$	4n
